# Completion Rate and Satisfaction With Online Computer-Assisted History Taking Questionnaires in Orthopedics: Multicenter Implementation Report

**DOI:** 10.2196/60655

**Published:** 2024-11-13

**Authors:** Casper Craamer, Thomas Timmers, Michiel Siebelt, Rudolf Bertijn Kool, Carel Diekerhof, Jan Jacob Caron, Taco Gosens, Walter van der Weegen

**Affiliations:** 1 Research and Development Department, Interactive Studios, 's-Hertogenbosch, Netherlands; 2 IQ Health Science Department, Radboud University Medical Center, Kapittelweg 54, Nijmegen, 6525, Netherlands, 31 243615305; 3 Department of Orthopedic Surgery, Sports & Orthopedics Research Centre, Anna Hospital, Geldrop, Netherlands; 4 Department of Orthopaedics, Elisabeth Tweesteden Hospital, Tilburg, Netherlands; 5 Department of Medical and Clinical Psychology, Tilburg University, Tilburg, Netherlands

**Keywords:** computer-assisted history taking, history taking, digital medical interview, orthopedics, digital health, computer-assisted, cohort study, orthopedic, outpatient, satisfaction, patient engagement, medical record

## Abstract

**Background:**

Collecting the medical history during a first outpatient consultation plays an important role in making a diagnosis. However, it is a time-consuming process, and time is scarce in today’s health care environment. The computer-assisted history taking (CAHT) systems allow patients to share their medical history electronically before their visit. Although multiple advantages of CAHT have been demonstrated, adoption in everyday medical practice remains low, which has been attributed to various barriers.

**Objective:**

This study aimed to implement a CAHT questionnaire for orthopedic patients in preparation for their first outpatient consultation and analyze its completion rate and added value.

**Methods:**

A multicenter implementation study was conducted in which all patients who were referred to the orthopedic department were invited to self-complete the CAHT questionnaire. The primary outcome of the study is the completion rate of the questionnaire. Secondary outcomes included patient and physician satisfaction. These were assessed via surveys and semistructured interviews.

**Implementation (Results):**

In total, 5321 patients were invited, and 4932 (92.7%) fully completed the CAHT questionnaire between April 2022 and July 2022. On average, participants (n=224) rated the easiness of completing the questionnaire at 8.0 (SD 1.9; 0‐10 scale) and the satisfaction of the consult at 8.0 (SD 1.7; 0‐10 scale). Satisfaction with the outpatient consultation was higher in cases where the given answers were used by the orthopedic surgeon during this consultation (median 8.3, IQR 8.0‐9.1 vs median 8.0, IQR 7.0‐8.5; *P*<.001). Physicians (n=15) scored the average added value as 7.8 (SD 1.7; 0‐10 scale) and unanimously recognized increased efficiency, better patient engagement, and better medical record completeness. Implementing the patient’s answers into the electronic health record was deemed necessary.

**Conclusions:**

In this study, we have shown that previously recognized barriers to implementing and adapting CAHT can now be effectively overcome. We demonstrated that almost all patients completed the CAHT questionnaire. This results in reported improvements in both the efficiency and personalization of outpatient consultations. Given the pressing need for personalized health care delivery in today’s time-constrained medical environment, we recommend implementing CAHT systems in routine medical practice.

## Introduction

### Background

The patient’s medical history plays a crucial role in establishing an accurate diagnosis [1-3]. However, collecting the medical history during a first consultation is time-consuming, and time is scarce in today’s health care environment. In addition, the first consultation can be a stressful event for a patient, resulting in anxiety and misinterpretation of the questions asked during a medical encounter [4]. Subsequently, this can potentially result in incomplete and invalid information, hindering a patient’s ability to participate in shared decision-making [4].

Computer-assisted history taking (CAHT) systems, also known as digital medical interview assistant systems, are software programs that allow patients to present their medical history electronically before an outpatient consultation. For instance, this can be done remotely via a web-based portal or smartphone app prior to the scheduled consultation [5]. CAHT was first introduced in the early 1970s as an additional channel to collect highly relevant, comprehensive, and accurate patient information [6]. Multiple advantages of CAHT have been demonstrated, including saving face-to-face consultation time spent on history taking and empowering patients to be active in their own care [7]. Moreover, CAHT might enhance the comprehensiveness of patient history taking by employing standardized algorithms that expand questioning depth based on the participant’s responses [8]. This approach holds the potential to uncover psychosocial and psychiatric issues potentially associated with the presenting complaint [9].

Although these findings are promising, the adoption rate of CAHT within health care remains low. This is attributed to various barriers for both health care professionals (HCPs) and patients [6]. The accessibility of health care for all comes into question while digitalizing health care. Additionally, concerns arise regarding the interoperability of data that is fragmented across multiple compartments. The ability of patients to provide accurate answers is also brought into focus when they are consulted via an online survey rather than an in-person consultation [10].

Despite these barriers in integrating CAHT into everyday medical practice, the current pressure on the health care system demands action. Since the projected growth of multiple patient populations by far exceeds the number of available HCPs in the near future, the time spent on each patient needs to be as efficient and effective as possible, without reducing (perceived) health care quality. Given the number of patients that nowadays have access to email, websites, and smart devices, and the unprecedented advances in technologies in recent years, a more successful implementation of CAHT in clinical practice could be expected. However, no research has been published about achieving this goal.

### Objectives

The aims of this study were to implement an online orthopedic CAHT questionnaire that enables patients to provide their medical history prior to their first outpatient consultation and to integrate the CAHT system into the electronic health records (EHRs) of two Dutch hospitals. We subsequently analyzed the completion rate of the CAHT questionnaire, as well as HCPs’ satisfaction with the collected information, its accessibility, and its added value.

## Methods

### Study Design and Setting

This multicenter implementation study was conducted at the orthopedic departments of the Anna Hospital (Geldrop, The Netherlands) and Elisabeth-Tweesteden Hospital (Tilburg, The Netherlands). No changes were made to the design after the study was commenced. We followed the implementation guidelines for the reporting on digital health implementations [11].

### Ethical Considerations

Approval was obtained from the medical ethics committees of Anna Hospital and Elisabeth-Tweesteden Hospital. The study was exempted from the Medical Research Involving Human Subjects Act (WMO, N23.090). Patients were informed about data usage for research and publication purposes at the start of the CAHT questionnaire, with participation being voluntary.

### Participant Selection

All patients aged 18 years and older who were referred to the orthopedic departments of the participating centers for their first in-hospital consultation were invited to participate in the study. Patients needed to have an email address and sufficient command of the Dutch language. Patients with a cognitive disorder and patients specifically referred for pediatric orthopedics were excluded. Inclusion criteria were assessed by hospital staff when they contacted the patients to schedule their appointment.

All physicians and orthopedic residents (n=24) working in the participating hospitals and who have had scheduled initial consultations with patients during the study period were invited to participate in the study as well [12].

### The CAHT Questionnaire

**Figure 1.**
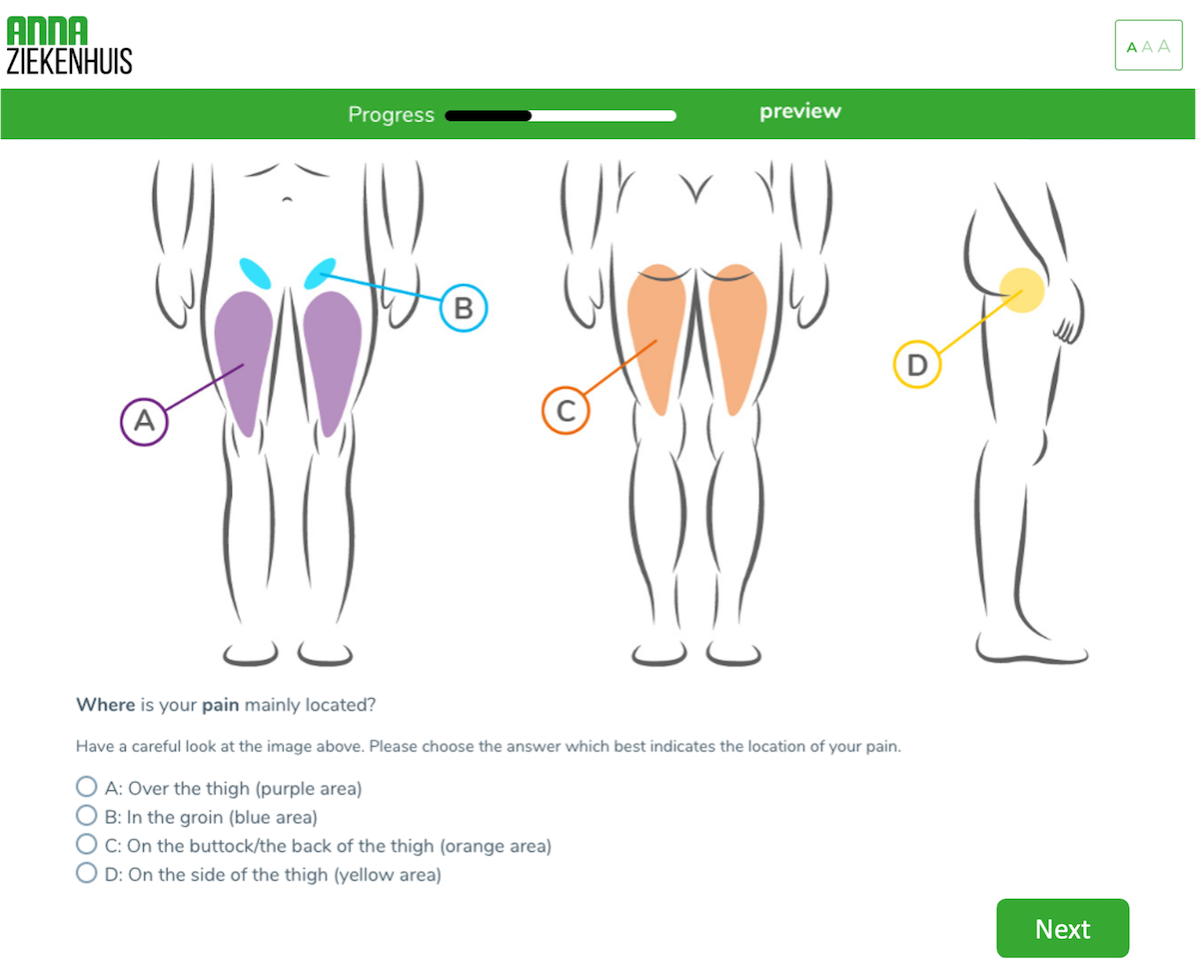
Example of the computer-assisted history taking questionnaire for patients with hip complaints. Patients can indicate the location of their hip pain using an illustration. Questions are translated from Dutch (the language used in the study) to English.

The CAHT questionnaire aimed to collect the patients’ medical condition, in preparation for their first outpatient consultation. The questionnaire included several topics in the following order: affected joint, previous diagnoses or treatments, health status, personal care needs or preferences, and patient characteristics (Table 1). Some questions were generic, but most were joint-specific. Depending on the answers given by the patient on specific questions, standardized algorithms expand questioning depth using branching logic without using artificial intelligence (eg, in the case of trauma, more information was requested about the trauma origin). The online questionnaire was offered to patients in a design consistent with the hospital’s branding, and some questions were supported with illustrations and instructions (Figure 1).

**Table 1.** Example of covered subjects and related questions for patients with knee complaints. Questions are translated from Dutch (the language used in the study) to English.

Topic	Question
History	Have you ever received a diagnosis**,** by a physician or general practitioner, due to complaints in your knee and/or your lower back? Have you ever undergone a surgical procedure for your knee and/or lower back?
Main complaint	For which knee do you have complaints? For how long have you experienced knee complaints? On a scale of 0 to 10, how severe is your knee pain at rest?
Main complaint in relation to social activities	Are you limited in playing sports or executing your hobby due to your knee complaints? Are you limited in your job due to your knee complaints?
Personal care needs and preferences	Your orthopedic surgeon would like to know what your main worry or question is regarding your complaints. This way the consultation can be about what’s important to you. If necessary, to what extent are you willing to undergo a surgical procedure to get rid of your knee complaints?
Effect of conservative therapies	Have you tried muscle-strengthening physical training for a period of 4 to 6 weeks?

### Development of the CAHT Questionnaire

**Figure 2.**
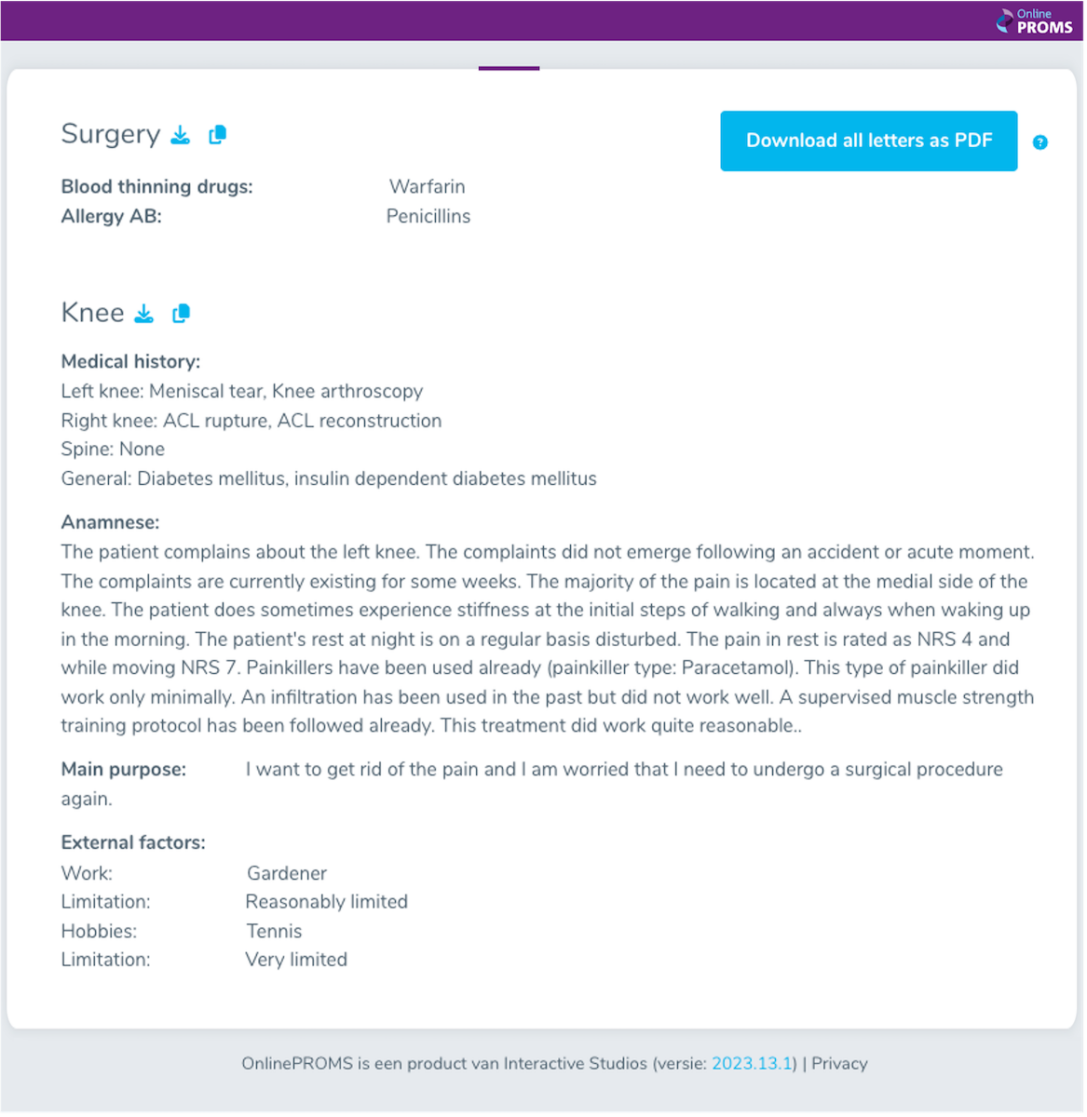
Example of the answers to the computer-assisted history taking questionnaire, presented as a coherent summary of a patient indicating the knee as the affected joint. The summary is translated from Dutch (the language used in the study) to English. AB: antibiotics; ACL: anterior cruciate ligament; NRS: numeric rating scale.

The CAHT questionnaire and its output were developed between January 2022 and March 2022. An expert panel was created for the development of the CAHT questionnaire and its output and consisted of three experienced orthopedic surgeons from both hospitals. First, the input from the physicians at Anna Hospital was obtained, offering insights from the perspective of their professional focus area. Subsequently, the draft version was developed and internally reviewed before undergoing external assessment by three orthopedic surgeons from the Elisabeth Tweesteden Hospital. The answers to the CAHT questionnaire were automatically presented as a coherent summary in a format designed by the expert panel, without providing a differential diagnosis (Figure 2). A web link from the CAHT platform with single sign-on was integrated with the two EHRs used by the participating hospitals in March 2022. This allowed the physicians to read and interpret the data, and to alter it when deemed necessary during the consultation. All feedback on the questionnaire and its output were processed before the study commenced.

### The CAHT Platform

**Figure 3.**
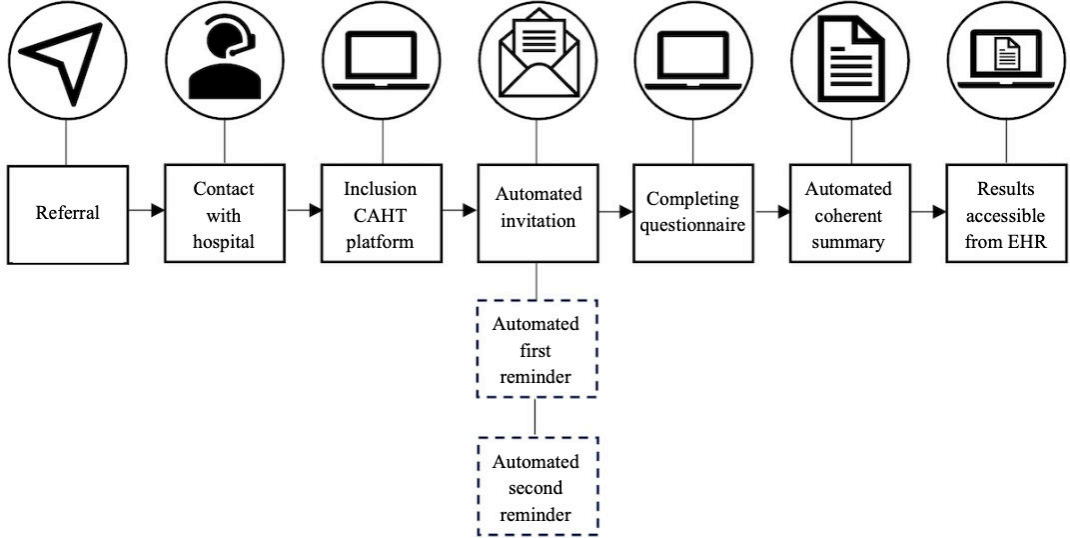
Flowchart of the computer-assisted history taking (CAHT) platform. After a patient was referred to the hospital and got in contact with the hospital staff, patients were included, and subsequently, an automated invitation was sent. If the patient did not respond to the invitation, a first and a second reminder were sent based on the response schedule. If the patient completed the questionnaire, a coherent summary of the answers was generated automatically from the available data and was eventually accessible from the electronic health record (EHR).

OnlinePROMS (Interactive Studios, ‘s-Hertogenbosch, The Netherlands) was used as the CAHT platform. Interactive Studios has been active in the Dutch health care market for over a decade, providing sustainable solutions for remote patient monitoring. The platform meets European regulations for the privacy and security of patient-reported health data. Invitations to answer the CAHT questionnaire were automatically sent by email when a patient was added to the platform by hospital administration staff. The CAHT questionnaire became accessible to the patient after a two-factor authentication code was entered. The platform allowed hospitals to send two reminders by either email or SMS text messaging if the patient did not complete the questionnaire. Participants of Anna Hospital were reminded 1 and 2 days after the initial invitation at 9 AM via SMS text messaging or email if the phone number was unknown. The ETZ Hospital was chosen to send automated reminders 5 and 10 days after the initial invitation at 9 AM (Figure 3).

### Study Outcomes

The percentage of patients who completed the CAHT questionnaire was the primary outcome. The usage statistics were collected as secondary outcomes, including the time needed to complete the CAHT questionnaire and the number of necessary reminders. Patient demographic data were collected from the CAHT questionnaire (age, sex, BMI, and affected joint). In addition, in July 2022, all patients were invited to answer a questionnaire on the usage of the CAHT questionnaire. After reaching a convenience sample (n=224), this invitation was deactivated. All physicians of the participating hospitals were asked to rate their satisfaction with the information collected with the CAHT system as well as its accessibility and added value. An overview of the study outcomes is presented in Table 2. All data were collected for the duration of the study via the OnlinePROMS platform.

**Table 2.** Overview of the study outcomes.

Outcome	Description
Completion percentage^a^	The percentage of patients who completed the CAHT^b^ questionnaire in preparation for their outpatient consultation.
Duration to complete^c^	Time (in minutes) needed to complete the CAHT questionnaire.
First reminders sent^c^	The number of first reminders automatically sent by the CAHT platform in case the CAHT questionnaire was not completed yet.
Second reminders sent^c^	The number of second reminders automatically sent by the CAHT platform in case the CAHT questionnaire was not completed yet.
Patient demographics^a^	Age, sex, BMI, and affected joint.
Easiness of the CAHT questionnaire^d^	Easiness of the CAHT questionnaire on a 10-point Likert scale.
Patient satisfaction^d^	Satisfaction of the consultation on a 10-point Likert scale.
Usage by the physician^d^	The number of patients reporting that the CAHT questionnaire summary was used by the physician during consultation.
Added value^d^	The added value of a CAHT questionnaire supported medical history taking during consultation on a 10-point Likert scale.
HCP^e^ data accessibility^f^	Experienced easiness for data accessibility. Satisfaction is rated on a 10-point Likert scale.
Added value for HCP^f^	The added value of a completed CAHT questionnaire during outpatient consultation on a 10-point Likert scale.

a Collected prior to consultation.

b CAHT: computer-assisted history taking.

c Derived from platform user statistics.

d Collected 1 day after consultation.

e HCP: health care professional.

f Collected at the end of the study.

### Statistical Methods

Categorical variables are presented as numbers and percentages. Normally distributed continuous variables are presented as means (with the SD). Nonnormally distributed variables are presented as the median value (with the IQR). To analyze satisfaction between groups, data were considered statistically significant at *P*<.05. In case of normal distribution and variances, an independent *t* test was used. For nonnormal distributions, a nonparametric test was used. All data was analyzed using IBM SPSS Statistics for Macintosh, version 29.0 (IBM Corp).

### Budget Planning

The license fee of the CAHT platform was estimated to be €1000 (US $1085) per month regardless of the number of patients included and health care providers involved. In addition, a one-time setup fee for the questionnaire was estimated to be €4000 (US $4343). Finally, a one-time setup fee for the integration with the EHR was estimated to be €4000 (US $4343).

## Implementation (Results)

### Study Sample

**Figure 4.**
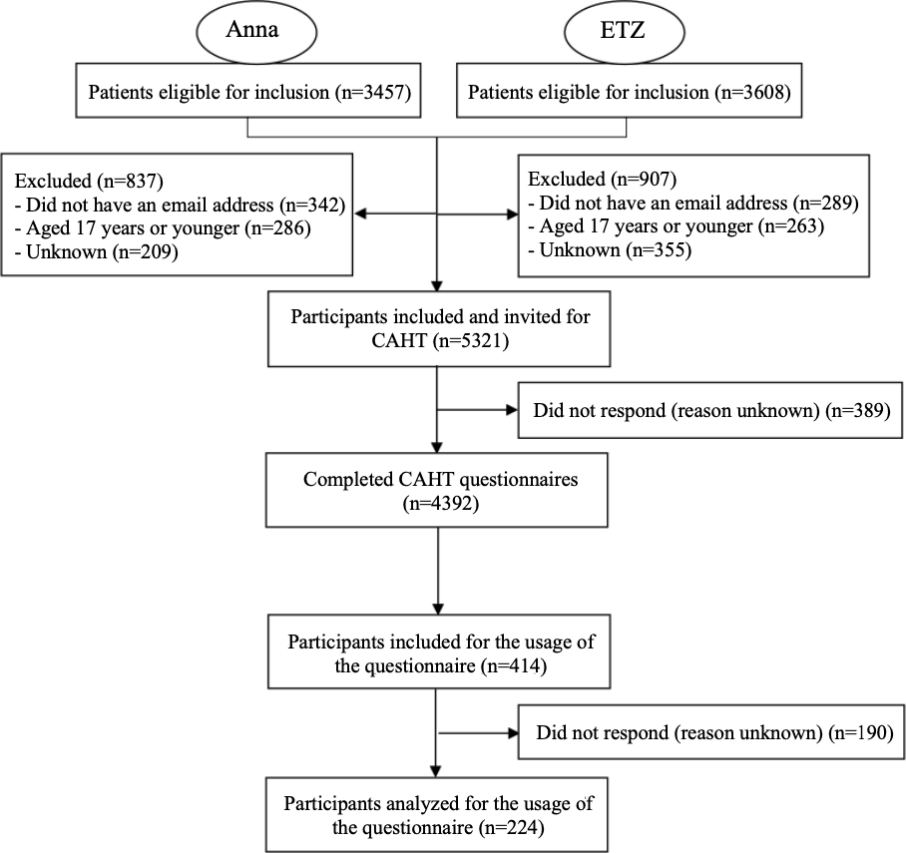
Flow diagram showing the inclusion and exclusion of patients. Anna: Anna Hospital; CAHT: computer-assisted history taking; ETZ: Elisabeth Tweesteden Hospital.

Between April 2022 and July 2022, a total of 7065 patients were scheduled to have a first consultation with an orthopedic surgeon in 1 of the 2 participating hospitals. Of these, 5321 (75.3%) met the study’s inclusion criteria. In addition, a usability questionnaire was sent to 414 participants, of whom 224 (54.1%) responded (Figure 4). Semistructured interviews were performed with 15 orthopedic surgeons from the participating hospitals.

### Completed CAHT Questionnaires

In total, 5321 patients were invited to complete the CAHT questionnaire. Out of these, 4932 (92.7%) participants fully completed the questionnaire. Anna Hospital invited 2620 patients, of whom 2516 (96.0%) completed the CAHT questionnaire. ETZ Hospital invited 2701 patients, of whom 2416 (89.4%) completed the CAHT questionnaire (Table 3).

**Table 3.** Number of invitations sent and completed computer-assisted history taking questionnaires, divided by hospital.

	Invitations sent, n (%)	Completed, n (%)
Anna Hospital	2620 (49.3)	2516 (96.0)
Elisabeth Tweesteden Hospital	2701 (50.8)	2416 (89.4)
Total	5321 (100)	4932 (92.7)

### Usage Statistics

**Figure 5.**
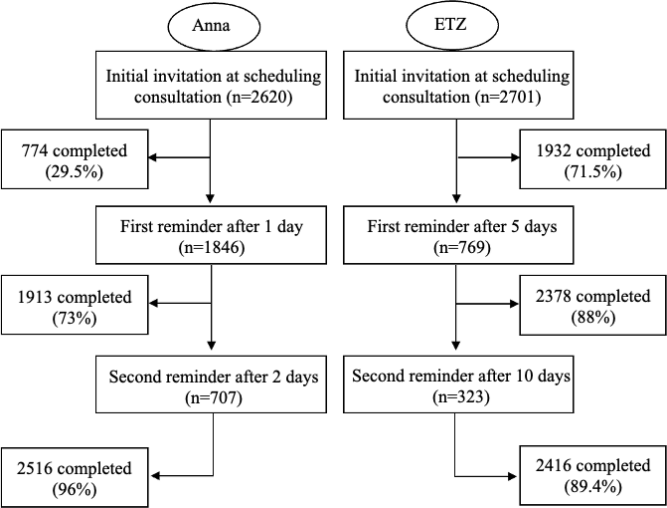
Flow diagram showing the response to automated sent invitations and reminders. Anna: Anna Hospital; ETZ: Elisabeth Tweesteden Hospital.

The duration to complete the questionnaire for participants who started and completed the questionnaire on the same day displayed a median value of 16.2 (IQR 11.4‐26.1) minutes. In addition to the initial invitation, Anna Hospital needed 1846 first and 707 second reminders and ETZ needed 796 first and 323 second reminders, respectively (Figure 5).

### Patient Characteristics

Table 4 shows the patient characteristics. The mean participant age was 56.5 (SD 16.8) years, and 2208 out of 4932 (44.8%) participants were men with a mean BMI of 27.4 (SD 5.1) kg/m^2^. The three joints most often selected as affected by the 4932 participants were the knee (n=2205, 44.7%), hip (n=962, 19.5%), and ankle/foot (n=908, 18.4%).

**Table 4.** Participant characteristics.

Characteristic	Patients from Anna Hospital (n=2516)	Patients from Elisabeth Tweesteden Hospital (n=2416)
Age (years), mean (SD)	55.4 (16.9)	57.5 (16.7)
18‐27, n (%)	239 (9.5)	165 (6.8)
28‐37, n (%)	206 (8.2)	190 (7.9)
38‐47, n (%)	254 (10.1)	272 (11.3)
48‐57, n (%)	548 (21.8)	468 (19.4)
58‐67, n (%)	592 (23.5)	552 (22.8)
68‐77, n (%)	511 (20.3)	529 (21.9)
78‐87, n (%)	156 (6.2)	220 (9.1)
88‐97, n (%)	10 (0.4)	20 (0.8)
Sex (male), n (%)	1157 (46)	1051 (43.5)
BMI (kg/m^2^), mean (SD)	27.2 (16.8)	27.6 (5.2)
Affected joint^a^, n (%)		
Knee	1205 (47.9)	1000 (41.4)
Hip	437 (17.4)	525 (21.7)
Ankle/foot	423 (16.8)	485 (20.1)
Shoulder	409 (16.3)	310 (12.8)
Elbow	34 (1.4)	48 (2.0)
Wrist/hand	57 (2.3)	116 (4.8)
Spine	183 (7.3)	349 (14.4)

a The computer-assisted history taking questionnaire allowed patients to select multiple affected joints.

### Patient Satisfaction

Participants included were asked to rate the CAHT questionnaire usability (n=224); on average, they rated it an 8.0 (SD 1.9) out of 10 on easiness to complete the questionnaire and an 8.0 (SD 1.7) out of 10 considering the satisfaction of the consult. Satisfaction with the outpatient consultation was higher in cases where the given answers were used by the orthopedic surgeon during this consultation (median 8.3, IQR 8.0‐9.1 vs median 8.0, IQR 7.0‐8.5; *P*<.001). Out of 224 participants, 145 (64.7%) reported that the CAHT questionnaire was used by the physician during the consultation and 157 out of 224 (70.4%) participants had the feeling that their physician had a better understanding of their complaint due to the CAHT questionnaire.

### Physician Satisfaction

On average, physicians (n=15) scored the added value of using the CAHT questionnaires during their consultation as 7.8 (0‐10 scale, SD 1.7). One physician reported not using the answers at all during the consultation. Physicians’ unanimously recognized benefits during outpatient consultations, such as increased efficiency, better patient engagement, and better medical record completeness. This was more the case in patients with hip or knee complaints, and less in those with foot and ankle complaints, most likely due to the foot and ankle’s more complex anatomy, making it harder for patients to pinpoint the exact location of their symptoms. Physicians highlighted the questionnaire’s value in eliciting pertinent information, aiding diagnosis, and providing a framework for informed decision-making. Implementing the summary generated from patients’ answers in the EHR was deemed necessary to achieve this with direct access to the information from their own workspace.

## Discussion

### Principal Findings

This study demonstrates the feasibility of the implementation and clinical adaptation of CAHT for orthopedic patients scheduled for their first consultation in a hospital. The completion rate to answer a CAHT questionnaire before the first consultation was very high: 4932 out of 5321 (92.7%). Patients found the questionnaire easy to understand and complete. Additionally, they were more satisfied with their outpatient consultation when the summary of the CAHT questionnaire was taken into consideration by their physician. Physicians rated the CAHT questionnaire to be a useful addition to standard outpatient consultations, as insight into the personal care needs and preferences allowed them to address the main concern of the patient directly.

To achieve a high completion rate of the CAHT questionnaire, we took implementation barriers addressed in previous studies (accessibility, accuracy, and acceptability) into consideration [6]. For accessibility, linked with interoperability [13], we integrated a single sign-on web link that displayed a comprehensive summary (and the entire questionnaire when needed) of the answers directly in the EHR. From a patient perspective, the CAHT questionnaire was easily accessible from their email inbox with a two-factor authentication code. Regarding the accuracy, there is conflicting evidence [10,14]. We aimed to obtain accurate answers by enriching some questions with illustrations or a short instruction and using easy/informal language where possible. For data acceptance [15], we created an expert panel to develop and design the CAHT questionnaire and its output. This method is underlined by research, addressing the improvement in quality and acceptance of data [16].

Examining the impact resulting from the implementation of CAHT in a multicenter study design represents a major strength of this study. By incorporating 2 hospitals (1 nonacademic teaching hospital and 1 general hospital), we were able to include a high number of patients, strengthening the generalizability and robustness of our findings. This is confirmed by the similarity of the data between hospitals in terms of demographics, completion rate, and satisfaction.

This study is not without limitations. In this study, we implemented CAHT within orthopedic departments only, limiting the generalizability to other medical departments. The study population’s age and sex distribution might, however, indicate usability within other departments as well. Another limitation is the absence of nonverbal communication that occurs in face-to-face conversations. In contrast, CAHT completion allows for more time to think about the answers and fact-check them, without the stress and shortage of time experienced during outpatient visits. The reported median time of 16 minutes for completion supports this.

### Lessons Learned

Our study demonstrates that implementing CAHT in the daily routine of an orthopedic department is feasible and can lead to good clinical adaptations but does require the necessary steps to be taken. Requirements are that the patients have an email address, and hospital staff must be available to invite patients to the CAHT platform. The latter would ideally be done through an automated connection with the EHR. Making the results of the questionnaire available in the EHR can be done through a single sign-on web link, offered by almost all EHR suppliers. In addition, the development of the CAHT questionnaire by an expert panel was considered important to ensure usability for HCPs. The integration of the CAHT system with the EHRs and the presentation of the CAHT questionnaire answers as a coherent summary in a format designed by the expert panel were crucial elements for its adoption as well. We hypothesize that key factors contributing to a high completion rate among patients include the predefinition of questions in a standardized order, the inclusion of smart dependencies to avoid unnecessary questions, and the presentation of the online questionnaire in a design consistent with the hospital’s branding.

### Future Research

Today’s health care system is facing an immense burden. Time is limited, but personalized health care needs to be maintained or even improved. Increased consultation duration is associated with better health outcomes, fewer prescriptions, and better recognition of long-term and psychosocial problems [17,18], but this is simply impossible in most health care systems. Nevertheless, a physician who is supported by CAHT results might optimize consultation time in a friendly manner while improving patient-centered communication (ie, signposting, summarization, and repetition of the medical history) [19]. This may lead to more accurate diagnoses, enhanced shared decision-making, and increased patient satisfaction [20,21]. Future research should aim to study the effect of optimized face-to-face consultation time with the support of CAHT and its effect on satisfaction and cost-effectiveness.

### Conclusion

Previously reported barriers to implementing and adapting CAHT in clinical practice can nowadays be resolved. In this study, we demonstrated that almost all patients completed the CAHT questionnaire before their outpatient consultation. Both patients and HCPs reported a more efficient and personalized consultation when the answers to the questionnaire were used. Given the pressing need for personalized health care delivery in today’s time-constrained medical environment, we recommend implementing CAHT systems in routine medical practice.

## Abbreviations

CAHT: computer-assisted history taking
EHR: electronic health record
HCP: health care professional
